# Development of Genetic Modification Tools for *Hanseniaspora* *uvarum*

**DOI:** 10.3390/ijms22041943

**Published:** 2021-02-16

**Authors:** Jennifer Badura, Niël van Wyk, Silvia Brezina, Isak S. Pretorius, Doris Rauhut, Jürgen Wendland, Christian von Wallbrunn

**Affiliations:** 1 Department of Microbiology and Biochemistry, Hochschule Geisenheim University, Von-Lade-Strasse 1, 65366 Geisenheim, Germany; jennifer.badura@hs-gm.de (J.B.); niel.wyk@hs-gm.de (N.v.W.); silvia.brezina@hs-gm.de (S.B.); doris.rauhut@hs-gm.de (D.R.); juergen.wendland@hs-gm.de (J.W.); 2 ARC Centre of Excellence in Synthetic Biology, Department of Molecular Sciences, Macquarie University, Sydney, NSW 2113, Australia; sakkie.pretorius@mq.edu.au

**Keywords:** *Hanseniaspora uvarum*, transformation, genetic modification, *ATF1*, ethyl acetate, fermentation

## Abstract

Apiculate yeasts belonging to the genus *Hanseniaspora* are commonly isolated from viticultural settings and often dominate the initial stages of grape must fermentations. Although considered spoilage yeasts, they are now increasingly becoming the focus of research, with several whole-genome sequencing studies published in recent years. However, tools for their molecular genetic manipulation are still lacking. Here, we report the development of a tool for the genetic modification of *Hanseniaspora uvarum*. This was employed for the disruption of the *HuATF1* gene, which encodes a putative alcohol acetyltransferase involved in acetate ester formation. We generated a synthetic marker gene consisting of the *HuTEF1* promoter controlling a hygromycin resistance open reading frame (ORF). This new marker gene was used in disruption cassettes containing long-flanking (1000 bp) homology regions to the target locus. By increasing the antibiotic concentration, transformants were obtained in which both alleles of the putative *HuATF1* gene were deleted in a diploid *H*. *uvarum* strain. Phenotypic characterisation including fermentation in Müller-Thurgau must showed that the null mutant produced significantly less acetate ester, particularly ethyl acetate. This study marks the first steps in the development of gene modification tools and paves the road for functional gene analyses of this yeast.

## 1. Introduction

The term apiculate yeast signifies a distinctive lemon-shaped cell structure of yeast species belonging to the genus of *Hanseniaspora* and closely-related genera, such as *Saccharomycodes* and *Nadsonia* [1]. The genus *Hanseniaspora* has a cosmopolitan distribution and has often been reported to be the most abundant genus on mature and intact grape berries, and thus contributes the largest yeast population to the initial stages of a grape must fermentation [2,3]. Since *Hanseniaspora* is prevalent on grapes in almost all vineyards worldwide [4,5,6,7,8], it plays a crucial role in affecting the sensory profile of wine, especially regarding the complexity of wine [9,10,11]. In general, *Hanseniaspora* populations decrease significantly during wine fermentation as these yeasts are usually outcompeted by the main wine yeast, *Saccharomyces cerevisiae*. It was initially thought that the gradual increase in ethanol concentration was primarily responsible for the decline in the *Hanseniaspora* population, but there is growing scientific evidence suggesting that a variety of metabolites secreted by *S*. *cerevisiae* significantly impacts the viability of *Hanseniaspora* within a grape must fermentation [12]. Moreover, *Hanseniaspora* strains have been shown to withstand ethanol concentrations of more than 10% and are able to produce a considerable amount of ethanol themselves [12,13,14]. In the past, *Hanseniaspora* populations have been regarded to be spoilage yeasts when a significant proportion of these apiculate yeasts persists within a fermentation, as some strains are capable of producing significant amounts of acetaldehyde, acetic acid, and ethyl acetate. Winemaking practices, such as the addition of SO_2_ or even the utilisation of yeasts, which produce killer toxins that have antimicrobial activity against apiculate yeasts [15], are used to limit the proliferation of *Hanseniaspora* yeasts. This hypothesis of *Hanseniaspora* as purely a spoilage yeast has been challenged and re-evaluated in recent years as wine researchers are reporting many beneficial oenological contributions that *Hanseniaspora* strains can provide when co-cultured with *S*. *cerevisiae* in a mixed-culture type of must fermentation. The oenological benefits of *Hanseniaspora* wine strains include lower final ethanol levels as well as increased acetate and ethyl ester concentrations [16,17,18]. Although selected *Hanseniaspora* strains have been used in mixed starter cultures with *S*. *cerevisiae* in wine fermentations, no *Hanseniaspora* strain is commercially available yet for use as an oenological starter culture [19]. Moreover, its ester production capability is particularly striking.

By sheer numbers found in fermentations, one of the most prolific species is that of *Hanseniaspora uvarum,* which, in the earlier literature, was also referred to as *Kloeckera apiculata*. Apart from its predominance and importance in winemaking, *H*. *uvarum* strains have also been isolated from many other fermented beverages, such as cider [20] or tequila [21], as well as from various food-related niches such as the processing of coffee beans [22] and cocoa fermentations [23] and from fresh-squeezed orange juice [24]. In addition to its presence in foods and beverages, *H*. *uvarum* has also been isolated from soil [25], plants [26], insects [27], birds [28], molluscs [29], shrimps [30] and snails [14]. Due to its antagonistic properties against mould development, *H*. *uvarum’s* potential as a biocontrol agent has also been investigated [31,32,33]. 

In spite of the increasing importance of *Hanseniaspora* in wine fermentations, only in the past couple of years, has large-scale genome sequencing provided information on *Hanseniaspora* species [34,35,36,37]; this wealth of information provides a basic framework for analysing the biology of *Hanseniaspora*. Recent research has shown that the low specific activity of pyruvate kinase, catalysing one of the rate limiting steps in the glycolytic pathway, could explain the reduced capacity of *Hanseniaspora* to form ethanol [38]. Analyses of different *Hanseniaspora* genomes have provided insight into an extensive loss of genes involved in cell cycle regulation and the maintenance of genome integrity [39]. Comparisons among different species and strains have revealed that *Hanseniaspora* isolates possess particular dynamic genome structures with various variations in ploidy [40].

A central element of gene-function analyses is the ability to generate deletion strains. However, there is still a lack of protocols for targeted gene deletions in *H*. *uvarum* based on DNA-mediated transformation to achieve homologous gene replacements with disruption cassettes. In this study, we developed a basic tool for the initiation of molecular characterisation of *H*. *uvarum* genes. To this end, we targeted the *H*. *uvarum ATF1* homolog encoding an alcohol acetyltransferase, successfully deleted both alleles in this diploid yeast, and compared the fermentation performance and flavour production of the mutant with the wild type.

## 2. Results

### 2.1. Plasmid Design and Construction

#### 2.1.1. Design and Testing of Promoters

**Figure 1:**
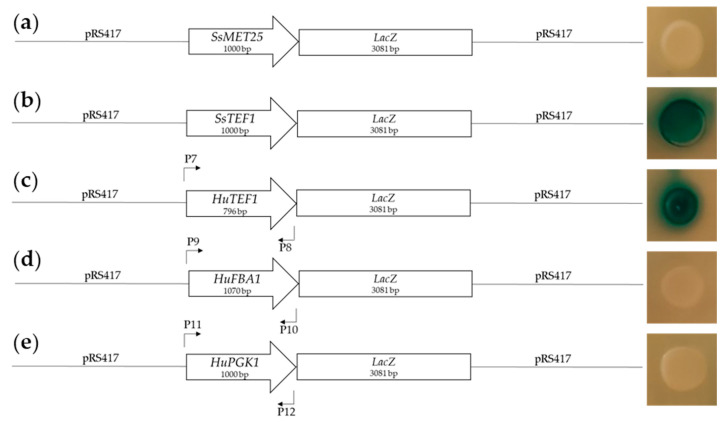
Plasmid constructs and testing of the functionality of three putative *Hanseniaspora uvarum* promoters, *HuTEF1*, *HuFBA1,* and *HuPGK1*, in *Saccharomyces cerevisiae* using *Streptococcus thermophilus LacZ* as reporter gene (picture on the right, blue colonies indicate the conversion of X-gal by beta-galactosidase). (**a**) pRS417-SsMET25-LacZ [41] (negative control); (**b**) pRS417-SsTEF1-LacZ [41] (positive control); (**c**) pRS417-HuTEF1-LacZ construct, generated as described in Section 4.2 using primer set 7/8 (for primers see description in Section 4.2; (**d**) pRS417-HuFBA1-LacZ, built using primer set 9/10; (**e**) pRS417-HuPGK1-LacZ built using primer set 11/12. *SsMET25* and *SsTEF1* are promoter sequences from *Saccharomycopsis schoenii* [41].

We originally attempted to transform *Hanseniaspora* using geneticin (*kanMX*) and hygromycin (*hygMX*) resistance cassettes already tailored for *S*. *cerevisiae* genetic modification. These cassettes are under the transcriptional control of the *TEF1* promoter of *Ashbya gossypii*. Despite numerous attempts to optimise transformation procedures, we were unable to obtain transformants and opted to clone promoters of *H*. *uvarum* to drive the expression of the respective antibiotic markers. To this end, we PCR-amplified three regions upstream of the open reading frames (ORFs) of *H*. *uvarum* genes whose *S*. *cerevisiae* homologs are known to be strongly expressed. These genes encode translation elongation factor EF-1α (*TEF1*), 3-phosphoglycerate kinase 1 (*PGK1*) and fructose-1,6-bisphosphate aldolase 1 (*FBA1*). We cloned these three *H*. *uvarum* promoters upstream of the *LacZ* reporter gene to test their heterologous function in *S*. *cerevisiae*. By transforming *S*. *cerevisiae* with these synthetic constructs, expression of beta-galactosidase can be monitored by its conversion of X-Gal into a blue strain. *S*. *cerevisiae* transformed with *HuFBA1* and *HuPGK1* constructs did not result in blue-stained *S*. *cerevisiae* colonies, suggesting that these promoters are non-functional in *S*. *cerevisiae*. However, use of the *HuTEF1* promoter yielded beta-galactosidase activity in *S*. *cerevisiae* (Figure 1). Next, we fused the *HuTEF1* promoter to ORFs of antibiotic resistance genes to generate new marker genes for *Hanseniaspora* and to drive transcription of these resistance markers in *Hanseniaspora* transformants.

#### 2.1.2. Choice of Candidate Gene and Design of the Knock-Out Cassette

**Figure 2:**
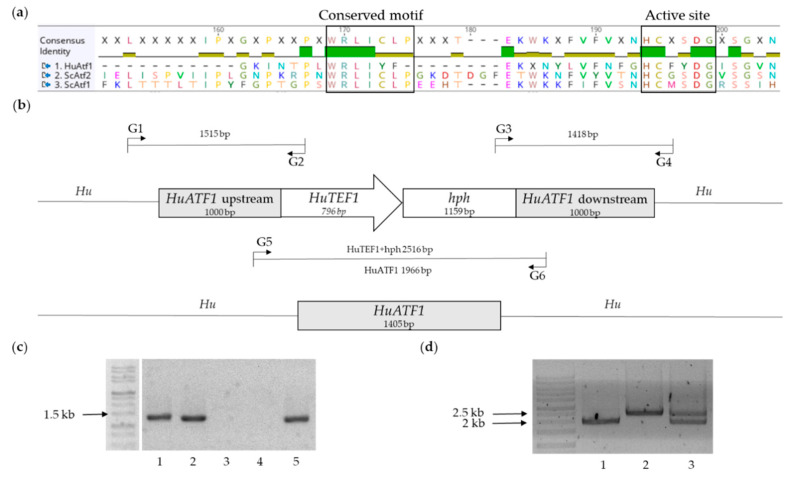
(**a**) Alignment of the amino acid sequence of the *Hanseniaspora uvarum* HuAtf1 with the two *S*. *cerevisiae* Atf proteins. The conserved WRLICLP motif as well as the HXXXDG active site [42] are shown in boxes; (**b**) Replacement of the *HuATF1* ORF by *HuTEF1_hph*. *HuATF1* was replaced by the *HuTEF1* promoter in front of the selection marker *hph* with 1000 bp of flanking regions that are upstream and downstream of the *HuATF1* ORF. The G1/G2, G3/G4, and G5/G6 diagnostic primer sets were used to verify correct integration of the marker cassette and deletion of *HuATF1*. The G5/G6 primer set was used to determine the absence of *HuATF1,* and thus a double knock-out due to the different nucleotide size of *HuATF1* and *HuTEF1*-*hph*; (**c**) GelRed stained agarose gel showing PCR amplification of *HuATF1* using primer set 39/40. (1) DSM2768 Ctrl, (2) DSM2768 #34 (WT/Δ), (3) DSM2768 #40 (Δ/Δ), (4) DSM2768 #46 (Δ/Δ), (5) DSM2768 #97 (WT/Δ). Resulting bands with size of ~1.4 kb; (**d**) GelRed stained agarose gel showing PCR amplification of wild type, single, or double knock-out of *HuATF1* using primer set 41/42 (G5/G6). (1) DSM2768 Ctrl, (2) DSM2768 #40 (Δ/Δ), (3) DSM2768 #34 (WT/Δ). Resulting bands with size of 2 kb and 2.5 kb.

We selected the *ATF1* alcohol acetyltransferase I gene from *H*. *uvarum* as the candidate gene to knock out, as *ATF1* and *ATF2* in *S*. *cerevisiae* are known to be the main catalysts for ethyl acetate production in wine. The generation of an *HuATF1* disruption cassette is described in the Material and Methods. The HuAtf1 protein shows only 22% homology to ScAtf1 and 25% ScAtf2, respectively. HuAtf1 contains both the active site HXXXDG [42] and the conserved WRLICLP motif of acetyltransferases (Figure 2a). The complete HuAtf1, ScAtf2, and ScAtf1 alignment can be seen in supplementary (Figure S1).

### 2.2. Generation of Strains and Verification of Transformants

We applied the two main *Saccharomyces* transformation procedures based on chemical or electro competence in order to transform *H*. *uvarum*. Although both methods yielded transformants, electroporation yielded more transformants. The specific electroporation protocol used, involved a sensitising step with lithium acetate [43]. We constructed selection cassettes containing the resistance markers for hygromycin (*hph*), geneticin (*neoR*), nourseothricin (clonNAT, *natI*), and zeocin (*Sh Ble*), commonly used for yeasts. The transformation of *H. uvarum* with the hygromycin-resistance selection cassette in the form of a linearised plasmid, containing *HuTEF1* and *hph*, each flanked by 1000 bp upstream and downstream homology regions of the ORF of *HuATF1*, yielded many transformants, without background colonies. In total, more than 100 colonies were re-streaked on selection plates, incubated overnight, and genomic DNA was isolated. Subsequently, PCR with diagnostic primers (G1/G2 and G3/G4, Figure 2b) was used to verify the correct intended integration of the selection cassette. In 99% of the colonies, the targeted insertion of the selection cassette could be detected via PCR. Integration of the cassette was also confirmed by sequencing of both the G1-G2 PCR product and G3-G4 PCR product. In addition, a second *HuATF1* allele was amplified by PCR suggesting a diploid state of *H*. *uvarum*.

Unfortunately, we were unable to obtain *H*. *uvarum* transformants using geneticin, nourseothricin, or zeocin in selection media. We found *H*. *uvarum* to be extremely resistant to these antibiotics even when we increased the concentration to two to three times more than what is recommended for the selection of *S*. *cerevisiae* transformants. This left us without a second dominant selection marker.

Since hygromycin proved to be the only antibiotic with which we were able to obtain *H*. *uvarum* transformants, we decided to transform it and plate the cells out on selective agar plates containing a three-fold higher concentration of hygromycin. From two out of 100 transformants that we obtained, we could not amplify an internal *HuATF1* band even after several PCR attempts, which suggested that both alleles of *ATF1* had been removed (Figure 2c). Primers were designed that bind outside the selection marker to the genomic DNA upstream and downstream of *HuATF1* (G5/G6, Figure 2b) and were used to amplify this sequence via PCR. GelRed stained agarose gel can be found in Figure 2d. While Samples 1 (DSM2768 Ctrl) and 2 (DSM2768 #40) show only one band each, with sizes of 2 kb (Sample 1) and 2.5 kb (Sample 2), two bands of both sizes are visible in Sample 3 (DSM2768 #34). In *H*. *uvarum* DSM2768, only the *HuATF1* band is visible. DSM2768 #34 showed two bands (*HuATF1* and the selection cassette) indicative of a heterozygous state (single knock-out). 

The transformant DSM2768 #40 (Sample 2) showed only one band corresponding to the size of the selection cassette, indicating an integration via homologous recombination. Sanger sequencing using primers G5 and G6 confirmed that only the *HuTEF1*-*hph* marker could be amplified from transformant DSM2768 #40. Accordingly, this is indicative of a homozygous deletion (double knock-out). Two independent homozygous deletion mutants were obtained (DSM2768 #40 and DSM2768 #46). The transformation efficiency of receiving double knock-out transformants obtained using the higher concentration of antibiotic was 2%.

### 2.3. Fermentations and Phenotypical Characterisation

**Figure 3:**
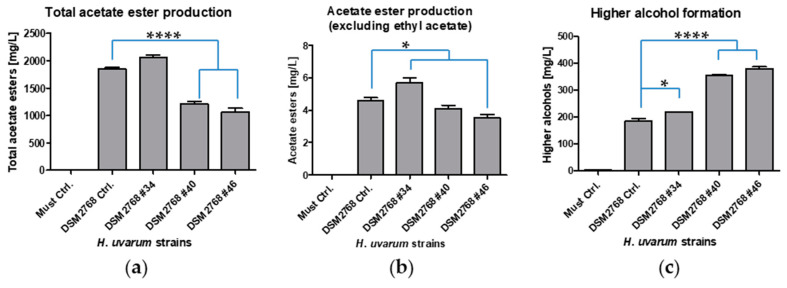
Acetate ester production and formation of higher alcohols during fermentation. (**a**) Total acetate ester production (mg/L) including ethyl acetate, isoamyl acetate, 2-methylbutyl acetate, hexyl acetate, and 2-phenylethyl acetate; (**b**) Acetate ester production (mg/L) excluding ethyl acetate; (**c**) The formation of respective higher alcohols (mg/L) including isoamyl alcohol, 2-methyl-1-butanol, hexanol, and 2-phenyl ethanol after three days of fermentation of Müller-Thurgau must with *H*. *uvarum* DSM2768 for comparison (DSM2768 Ctrl), DSM2768 #34 (WT/Δ), DSM2768 #40 (Δ/Δ), and DSM2768 #46 (Δ/Δ). Pure must without the addition of yeasts served as a negative control (Must Ctrl). Total ester production was measured via HS-SPME-GC-MS analysis while acetic acid production was measured via high performance liquid chromatography (HPLC) analysis. Data are the mean of three independent experiments ± SEM, 1-way ANOVA, Bonferroni’s multiple comparison test, * *p* < 0.05 and **** *p* < 0.0001. Error bars indicate the standard deviation.

**Figure 4:**
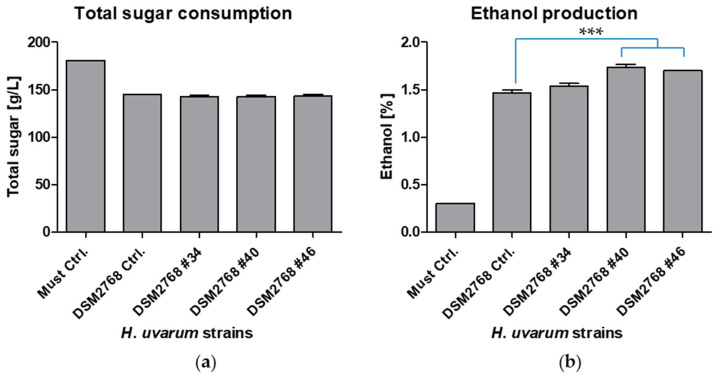
(**a**) Total sugar consumption (g/L); (**b**) Ethanol production (%) after three days of fermentation of Müller-Thurgau must with *H*. *uvarum* DSM2768 for comparison (DSM2768 Ctrl), DSM2768 #34 (WT/Δ), DSM2768 #40 (Δ/Δ), and DSM2768 #46 (Δ/Δ). Pure must without the addition of yeasts served as a negative control (Must Ctrl). The amount of total sugar and ethanol produced was measured via high performance liquid chromatography (HPLC) analysis. Data are the mean of three independent experiments ± SEM, 1-way ANOVA, Bonferroni’s multiple comparison test, *** *p* < 0.001. Error bars indicate the standard deviation.

In order to determine the phenotype of the transformants, fermentations with Müller-Thurgau must were performed. The phenotypical characterisation is shown in Figure 3. The most noteworthy difference between the double knock-out transformants (DSM2768 #40 and DSM2768 #46) and the control was the acetate ester content (Figure 3a). The most abundant acetate ester, ethyl acetate, had a concentration of 600–800 mg/L less in the Δ/Δ strains than the control (Supplementary Figure S2a). The four other acetate esters measured isoamyl acetate, 2-methylbutyl acetate, hexyl acetate, and 2-phenylethyl acetate also had significantly reduced amounts. In general, the alcohols of the respective esters showed an increase in the Δ/Δ strains, which also includes ethanol (Figure 3c and Figure 4b and Supplementary Figure S3). The Δ/Δ strains had an ~10% increase in ethanol. Interestingly, the Δ/Δ strain produced no detectable acetic acid and slightly increased levels of certain ethyl esters (ethyl propionate and ethyl butyrate) were found (Supplementary Figure S2f–h). The sugar consumption of all the strains was comparable over the period of three days (Figure 4a). The strain in which only one allele of *HuATF1* was removed performed similarly to the control strain apart from isoamyl acetate, 2-methylbutyl acetate, isobutanol, and 2-phenyl ethanol, which increased slightly in production as compared with the control (Supplementary Figures S2 and S3). On the basis of the observation that the putative double knock-out yeast strains metabolised the same amounts of sugars as the control strain, it was concluded that the transformants were a physiological fit.

## 3. Discussion

*Hanseniaspora uvarum* is an important yeast in the beverage industry, in particular for wine. In recent years, researchers have shown particular interest in this yeast, and reports on its beneficial impact on winemaking as well as several genome sequences have been published [34,35,36,37]. Despite the growing interest, to date, there are no genetic modification tools available for *H*. *uvarum*. 

Our study attempts to fill this gap and describes a successful genetic modification experiment in *H. uvarum*. A common antibiotic used in eukaryotic transformations, hygromycin, proved to be effective for selection purposes. Furthermore, *H*. *uvarum* was found not to be sensitive against other standard antibiotics which complicated our attempts to generate a double knock-out of the *HuATF1* gene. However, by increasing the hygromycin concentration, we were able to obtain colonies in which both alleles of *HuATF1* were successfully deleted. The transformation of *H*. *uvarum* DSM2768 with the linearised DNA containing the hygromycin selection cassette resulted in a highly efficient deletion of *HuATF1*. Previous reports have suggested that the phenotype of antibiotic resistance is dose dependent and that transformation efficiency correlates with the copy number in the yeast cell [44,45,46]. Hashida-Okado et al. [47] succeeded in generating a null mutant of a diploid *S*. *cerevisiae* strain by transforming with only one selection marker. These authors have shown that it is possible to disrupt both gene copies of homologous chromosomes of diploid cells in only one transformation step using one selection marker. After increasing the concentration of the antibiotic in the selective agar medium, transformants (such as DSM2768 #40) lacking *HuATF1* were obtained, and integration of the selection cassette could be verified. On the basis of these results, it was concluded that DSM2768 #40 (as well as DSM2768 #46) is a null mutant.

We showed that it was also possible to generate a double knock-out of *ATF1* in *H*. *uvarum* in such a fashion using only one selection cassette. This was done by tripling the antibiotic concentration in the selective plates. Here, the transformation efficiency of a single knock-out was 98% and that of a double knock-out was 2%.

The double knock-out stains showed a dramatic reduction in ethyl acetate production (~40%). *HuATF1* deletion did not completely abolish ethyl acetate production, suggesting other transferase enzymes to be involved in its production besides *HuATF1*. This is similar to *S*. *cerevisiae*, as up to half of the ethyl acetate is still present when the major *ATF* genes (*ATF1* and *ATF2*) are deleted, which are also known to be associated with ethyl acetate production [48,49].

The reduction in ethyl acetate was accompanied by an increase in the formation of ethanol. This can be explained by the function of *ATF1* to esterify ethanol to ethyl acetate. In the case of a deletion of *HuATF1*, this esterification of ethanol to ethyl acetate is restricted, resulting in an accumulation of ethanol during fermentation. The same applies to the other acetate esters and their respective alcohols. In addition to ethyl acetate, other acetate esters were also produced in smaller quantities by the null mutant strain. In contrast, the production of the corresponding alcohols increased significantly. Contrary to what we expected, significantly lower levels of acetic acid were produced by the double knock-out strains. This is in contrast to a previous study in *S*. *cerevisiae,* where no significant differences in acetic acid levels were detected in an *ATF1* knock-out strain as compared with the wild type [50]. Overexpression of *ATF1* in *S*. *cerevisiae* led to a decrease in acetic acid production [48,51], presumably as more of the available pool of acetyl coenzyme A (acetyl-CoA) is needed for the acetate ester synthesis. As a major catalyst for acetate ester production was removed in *H*. *uvarum*, we were not expecting a dramatic drop in acetic acid levels. This astonishing finding warrants further investigation. 

The knock-out of *HuATF1* did not lead to any apparent reduction in the vitality of *H*. *uvarum*. The null mutant strain metabolised the same amounts of total sugars as the control strain.

The development of suitable transformation tools for the widespread wine yeast *H*. *uvarum* enables more detailed investigations of the individual metabolic pathways for the formation of aroma compounds and the genes involved. In *S*. *cerevisiae*, these methods are common practice, and thus the individual gene functions and metabolic pathways are well characterised. Up to now, a detailed investigation of *H*. *uvarum* has not been possible. The next steps are the development of additional selection markers and the knock out or overexpression of other genes involved in the metabolism of the aroma compounds in *H*. *uvarum*.

## 4. Materials and Methods 

### 4.1. Strains and Culture Conditions

The yeast strains used and generated in this study (see Table 1) were routinely cultivated in YPD (1% yeast extract, 2% glucose, and 2% peptone) at either 25 °C (*H*. *uvarum*) or 30 °C (*S*. *cerevisiae*). Solid YPD plates were prepared by adding 2% agar and for transformation, two different concentrations of hygromycin b (200 and 600 µg/mL) were used. Plasmids were propagated in *Escherichia coli* DH5alpha in 2xYT (1.6% tryptone, 1% yeast extract, and 0.5% NaCl) supplemented with 100 µg/mL ampicillin.

### 4.2. Plasmid Design and Construction

Plasmids used in this study are listed in Table 2. Primers required for plasmid constructions are listed in Table 3. Plasmid constructions were assembled in *S*. *cerevisiae* BY4741 by yeast assembly. Plasmid DNAs were amplified in *E*. *coli* and prepared using a Plasmid Midi Purification Kit (Genaxxon, Ulm, Germany). For sequencing verification, PCR fragments were cloned either into pJET (Thermo Fisher Scientific, Waltham, MA, USA) or pGEM (Promega, Madison, WI, USA). DNA sequences of *H*. *uvarum* were obtained from the publicly available genomes of both DSM2768 (Accession: SAMN01885404) and AWRI3580 (Accession: SAMN04331434). By using corresponding *S*. *cerevisiae* homologs as queries, putative regions of interest, including the *ATF1* locus (*HuATF1*) and the promoter regions of *TEF1*, *FBA1,* and *PGK1* were identified using the BLAST function on NCBI. The *TEF1*, *FBA1,* and *PGK1* promoter were amplified from *H*. *uvarum* DSM2768 genomic DNA using primers 1/2 (*TEF1*), 3/4 (*FBA1*), and 5/6 (*PGK1*). To test for promoter activity, the *LacZ* ORF was placed under the control of *H*. *uvarum* promoters using primers 7/8 (*HuTEF1*-promoter), 9/10 (*HuFBA1*-promoter), and 11/12 (*HuPGK1*-promoter), which added 40 bp of flanking homology region to *pLacZ* (Figure 1). The upstream region of the *LacZ* ORF [41] was removed by restriction digestion of *pLacZ* with *Kpn*I and *Xho*I. The linearised vector and *H*. *uvarum* promoters (*HuTEF1*, *HuFBA1*, and *HuPGK1*) were transformed into yeast and fused to *LacZ* by *in vivo* recombination. Transformed cells were plated out on YPD plates +200 µg/mL geneticin. After two days of incubation, X-Gal was pipetted onto plates, and then plates were incubated to visualise beta-glucosidase activity. All restriction enzymes were purchased from Thermo Fisher Scientific (Waltham, MA, USA) and all primers were purchased from Sigma-Aldrich (Steinheim, Germany). The analyses of all PCR and restriction digestion products were performed by gel electrophoresis separation on 1% agarose gels, stained with GelRed (Genaxxon Bioscience, Ulm, Germany). Sequencing was conducted by Starseq, Mainz, Germany.

The transformation cassette for creating a null mutant of the *ATF1* gene within the genome of *H*. *uvarum* is shown in Figure 2b. For selection of transformants, the hygromycin-resistance gene ORF was placed under control of *HuTEF1* promoter. The pRS415 vector was linearised using *Bam*HI and *Hin*dIII. Flanking adaptor regions to control *in vivo* recombination were added to *HuTEF1* and *hph* marker fragments using the primer set 13/14 and 15/16. Next, BY4741 was transformed with the two PCR products and the cut vector. In addition, 1000 bp homology region upstream and downstream of *HuATF1* gene was amplified from *H*. *uvarum* DSM2768 genomic DNA using primers 17/18 and 19/20, containing 40 bp flanking homology region to pRS415 and either *HuTEF1* or *hph*. Flanking homology regions to guide *in vivo* recombination were added to pJB1-HuTEF-hph using primers 21/22. Then, BY4741 was transformed with the three PCR products and the linearised vector. Further knock-out cassettes with resistance genes against geneticin (*neoR*), nourseothricin (*natI*), and zeocin (*Sh Ble*) under the control of the *HuTEF1* promoter were designed similar to the hygromycin knock-out cassette. Primers 23–34 are additional primers that were specifically used to assemble the knock-out cassettes with these three antibiotic resistances.

To transform *H*. *uvarum* DSM2768, the constructed plasmid (pJB2-HuTEF-hph-ATF1up/down) was linearised using *Xho*I and *Xba*I. The cut plasmid was purified using GeneJET Gel Extraction Kit (Thermo Fisher Scientific, Waltham, MA, USA).

### 4.3. Yeast Transformation

The transformation of *S*. *cerevisiae* was done according to the lithium acetate/single strand DNA/polyethylene glycol 4000 protocol described by Gietz and Schiestl [57]. For transformation of *H*. *uvarum* DSM2768, an adaption of the protocols described by Thompson et al. [58] and Bernardi et al. [43] was used. *H*. *uvarum* DSM2768 was grown overnight in 5 mL YPD at 30 °C. Then, the culture was transferred into 50 mL YPD and incubated at 25 °C for 5 h. After centrifuging and washing with sterile double-distilled water, the culture was resuspended in 25 mL ice-cold 0.1 M lithium acetate/10 mM dithiothreitol/10 mM TE solution and incubated at room temperature for 1 h. The pellet was washed in 25 mL ice-cold sterile double-distilled water first, and then washed with 10 mL of ice-cold 1 M sorbitol. After centrifugation at 4 °C, the cells were resuspended in 100 µL of ice-cold 1 M sorbitol. For transformation, 100 µL of cell suspension were used per sample. An aliquot of 15 µL of transforming DNA or sterile distilled water as negative control, respectively, was mixed with the cell suspension and incubated on ice for 5 min before electroporation with 1.8 kV in 0.2 cm cuvettes. Cells were resuspended in 1 mL of ice-cold 1 M sorbitol and 300 µL YPD and transferred into a 1.5 mL reaction tube for a 3 h incubation at 30 °C. Shortly before the cells were plated out on selective plates (concentration of antibiotics: hygromycin 200–600 µg/mL, geneticin 200 µg/mL, nourseothricin 100–200 µg/mL, andzeocin 50–700 µg/mL) which did not contain sorbitol, they were centrifuged, and the cell pellet resuspended in 1 mL of YPD.

### 4.4. Verification of Yeast Transformants

Yeast transformants were picked, re-streaked on selection plates, and inoculated in 5 mL YPD including 200 µg/mL hygromycin. Genomic DNA was isolated from these cells by suspending a colony in a 100 μL solution of 200 mM lithium acetate and 1% sodium dodecyl sulphate and incubating in a 70 °C heat block, for three minutes. Thereafter, 300 μL of cooled 100% ethanol was added and centrifuged for 3 min at 13.000 min^−1^. The supernatant was discarded, then, 400 μL of cooled 70% ethanol was added and centrifuged again with the same settings. The supernatant was discarded, and the pellet was left to dry. Afterwards, the pellet was resuspended in 50 μL of TE buffer. For verification of transformation, G-primers were constructed (Figure 2b), targeting ~20 bp located within the transformation cassette (primers 36/37) and ~20 bp located within the genome of *H*. *uvarum* DSM2768 (primers 35/38). The resulting PCR product included either the 1000 bp *HuATF1* upstream or downstream region, respectively (primer sets used, 35/36 and 37/38).

### 4.5. Fermentations

In this study, pasteurised Müller-Thurgau grape must, harvested in 2019 from a vineyard of the Hochschule Geisenheim University in the Rheingau wine region of Germany, was used. The total sugar content was 180.9 g/L, of which 87.0 g/L was glucose and 93.3 g/L was fructose. The primary amino acid content of the must was analysed using the NOPA (nitrogen by o-phthaldialdehyde) assay and it amounted to 19.73 mg/L. The free ammonium level was 8.17 mg/L, as determined using a Rapid Ammonium kit from Megazyme (Bray, Ireland).

Fermentations with pasteurised Müller-Thurgau grape must using *H*. *uvarum* DSM2768, as well as the transformants *H*. *uvarum* DSM2768 (WT/Δ)/(Δ/Δ) generated within this study, were performed. Precultures of all yeast strains were prepared in shake flasks with YPD and incubated overnight at 30 °C. The next day, the must was inoculated with approximately 1 × 10^6^ cells/mL (as determined via hemocytometer). Fermentations were carried out in triplicate, utilising 250 mL fermentation flasks closed with aluminium foil. The fermentation temperature was 17 °C and flasks were shaken at 120 min^−1^ and fermentation was stopped after 3 days. Samples for high performance liquid chromatography (HPLC) analysis and volatile compound analysis (VCA) were taken and directly measured analytically. In addition, the bio-dry mass was determined.

### 4.6. High Performance Liquid Chromatography (HPLC) Analysis

The final concentrations of the major organic acids, sugars, and ethanol were determined by HPLC, using a method according to Schneider et al. [59] and modified as described in [60]. An HPLC Agilent Technologies Series 1100, equipped with a binary pump, an autosampler, a multi-wavelength detector (MWD), and a refractive index detector (RID, Agilent Technologies, Steinheim, Germany) was employed. A column with a length of 250 mm, an inside diameter of 4.6 mm, and a particle size of 5 µm was used for the measurements (Allure Organic Acids Column, Restek, Bad Homburg v. d. Höhe, Germany). The MWD was set at a wavelength of 210 nm for the detection of organic acids and the RID was used for the detection of carbohydrates, organic acids, and ethanol. The eluent used was double distilled water with 0.5% ethanol and 0.0139% concentrated sulfuric acid. The flow rate was 0.6 mL/min at a temperature of 46 °C.

### 4.7. Volatile Compound Analysis

To analyse volatile compounds, headspace-solid phase microextraction gas chromatography mass spectrometry (HS-SPME-GC-MS) analysis was applied according to Câmara et al. [61]. An amount of 5 mL of each sample was needed to measure the quantity of different aroma compounds. A GC 7890 A, equipped with a MS 5975 B (both Agilent, Santa Clara, CA, USA), and MPS robotic autosampler and CIS 4 (both Gerstel, Mülheim an der Ruhr, Germany), was used. Before the addition of 5 mL of wine sample and 10 µL of internal standard each (concentration of stock solution standards, 600 mg/L 1-octanol and 52 mg/L cumene), 1.7 g NaCl were weighed into a 20 mL headspace vial. Solid phase microextraction was carried out using a 65 μm polydimethylsiloxane and divinylbenzol fiber (Supelco, Merck, Darmstadt, Germany). Aroma compound separation was performed with a 60 m × 0.25 mm × 1 μm gas chromatography column (Rxi^®^-5Si1 MS w/5m Integra-guard, Restek, Bad Homburg v. d. Höhe, Germany) with helium as carrier gas. The sample was injected in split mode (1:10, initial temperature 30 °C, rate 12 °C/s to 240 °C, hold for 4 min). The gas chromatography (GC) run started with an initial temperature of 40 °C for 4 min, raised to 210 °C (5 °C/min), and then raised to 240 °C (20 °C/min) and held for 10.5 min. Mass spectral data were acquired in a range of mass to charge ratio (m/z) of 35 to 250 and used to derive concentration values. A 5-point calibration curve was used for each volatile compound within a wine model solution of 10% ethanol with 3% tartaric acid pH 3, as described previously [62].

### 4.8. Data Analysis

The HPLC data were analysed using the software ChemStation for LC systems from Agilent (Agilent, Santa Clara, CA, USA). The HS-SPME-GC-MS data analyses were carried out using Agilent’s MassHunter software (Agilent, Santa Clara, CA, USA).

The graphics were created with GraphPad Prism 5 (Graph Pad Software, La Jolla, CA, USA) and statistical analyses were employed in the form of a one-way analysis of variance (ANOVA) in combination with Bonferroni’s multiple comparison test. For statistical analysis, all fermentations were done in triplicate and the values for the metabolites are the means of these triplicates.

## Figures and Tables

**Table 1:** Strains used and generated in this study.

Strain	Feature/Genotype	Source
BY4741	*Saccharomyces cerevisiae MAT**a** his3Δ1, leu2Δ0, met15Δ0, ura3Δ0*	Euroscarf, Oberursel, Germany
DSM2768	*Hanseniaspora uvarum ATF1*/*ATF1*	[38]
DSM2768 (WT/Δ)	*Hanseniaspora uvarum ATF1*/*atf1*:*HuTEF1*-*hph*	This study
DSM2768 (Δ/Δ)	*Hanseniaspora uvarum atf1*:*HuTEF1*-*hph*/*atf1*:*HuTEF1*-*hph*	This study

**Table 2:** Plasmids used and generated in this study.

Strain	Feature/Genotype	Source
pRS415	*bla*, *LEU2*	[52]
pGEM	*bla*	Promega, Madison, WI, USA
pJET	*bla*	Thermo Fisher Scientific, Waltham, MA, USA
pRS417-SsTEF-lacZ	*bla*, *GEN3*, *lacZ* driven by *S*. *schoenii TEF1* promoter	[41]
pRS417-SsMET25-lacZ	*bla*, *GEN3*, *lacZ* driven by *S*. *schoenii MET25* promoter	[41]
pRS417-HuTEF-lacZ	*bla*, *GEN3*, *lacZ* driven by *H*. *uvarum TEF1* promoter	This study
pRS417-HuFBA-lacZ	*bla*, *GEN3*, *lacZ* driven by *H*. *uvarum FBA1* promoter	This study
pRS417-HuPGK-lacZ	*bla*, *GEN3*, *lacZ* driven by *H*. *uvarum PGK1* promoter	This study
pRS40H	*HygMX*	[53]
pTEF	pJET-*HuTEF1*	This study
pJB1-HuTEF-hph	pRS415-*HuTEF1*-*hph*	This study
pJB2-HuTEF-hph-ATF1up/down	pRS415-*HuTEF1*-*hph*-1000 bp *HuATF1* upstream/downstream	This study
pFA-KanMX6	*KanMX6*	[54]
pJB3-HuTEF-neoR	pRS415-*HuTEF1*-*neoR*	This study
pFA-NatMX3	*NatMX3*	[55]
pJB4-HuTEF-natI	pRS415-*HuTEF1*-n*atI*	This study
pZeo^R^	*BleMX6*	[56]
pJB5-HuTEF-Sh Ble	pRS415-*HuTEF1*-*Sh Ble*	This study
pJB6-HuTEF-Sh Ble-ATF1up/down	pRS415-*HuTEF1*-*Sh Ble*-1000 bp *HuATF1* upstream/downstream	This study

**Table 3:** Primers used in this study.

Primer Number	Primer Name	Sequence 5′ → 3′
**Hu-Promoter Test**		
1	TEF_L	TTGATGGATAACTTGAAGGC
2	TEF_R	TCTATATACTGTATACCTTAG
3	FBA_L	GAAGATATACTAAATTTGTCCC
4	FBA_R	AATGTATGTATTTGTATAATTGATATTATTATGG
5	PGK_L	TGATTATGACTCTGATAGCAAC
6	PGK_R	TTTTAAGATTTGTAGTAATTTAATTGTTTATATG
7	TEF forw. LacZ	CGCCAGGGTTTTCCCAGTCACGACGTTGTAAAACG ACGGCCAGTGTTGATGGATAACTTGAAGGC
8	TEF rev. LacZ	CAATCTTTGGATCGTTTAAATAAGTTTGAATTTTT TCAGTCATGTTTCTATATACTGTATACCTTAG
9	FBA forw. LacZ	CGCCAGGGTTTTCCCAGTCACGACGTTGTAAAACG ACGGCCAGTGGAAGATATACTAAATTTGTCCC
10	FBA rev. LacZ	CAATCTTTGGATCGTTTAAATAAGTTTGAATTTTTTCAGTCA TGTTAATGTATGTATTTGTATAATTGATAT TATTATGG
11	PGK forw. LacZ	CGCCAGGGTTTTCCCAGTCACGACGTTGTAAAAC GACGGCCAGTGTGATTATGACTCTGATAGCAAC
12	PGK rev. LacZ	CAATCTTTGGATCGTTTAAATAAGTTTGAATTTTTTCAGTC ATGTTTTTTAAGATTTGTAGTAATTTAATTGTTTATATG
**Construction Knock-Out Cassette** ***hph***		
13	pRS415 (ov) + TEF_L	ATTGGGTACCGGGCCCCCCCTCGAGGTCGACGGTA TCGATTTGATGGATAACTTGAAGGC
14	TEF_R+hyg (ov)	TTTTTCAACAGAAGTAGCAGTCAATTCTGGTTTTT TCATTCTATATACTGTATACCTTAG
15	TEF (ov) + hyg_L	AGCGGTATAACCATAGAAACTAAGGTATACAGTAT ATAGAATGAAAAAACCAGAATTGAC
16	hyg_R + pRS415 (ov)	GCTGGAGCTCCACCGCGGTGGCGGCCGCTCTAGAA CTAGTAGGACCACCTTTGATTGTAA
17	pRS415 (ov) + ATFup_L	ATTGGGTACCGGGCCCCCCCTCGAGGTCGACGGTA TCGATGTACATCTGTTTTTAATGCT
18	ATFup_R + TEF (ov)	AGTATGGCCATTGTTATGATGCCTTCAAGTTATCC ATCAACTCCGAGGTAATGTTTTTGA
19	Hyg (ov) + ATFdown_L NEW	AGGGTGGTAATTATTACTATTTACAATCAAAGGTG GTCCTTCAAAATATTGTATTTTCTT
20	ATFdown_R + pRS415 (ov) NEW	CTGGAGCTCCACCGCGGTGGCGGCCGCTCTAGAAC TAGTGTATCCCAACAAAAGATAGAA
21	ATFup (ov) + TEF_L	TTAACCCCAGCAGGAATACATCAAAAACATTACCT CGGAGTTGATGGATAACTTGAAGGC
22	Hyg_R + ATFdown (ov) NEW	TAAAAAGAATAAAACTTTGAAAGAAAATACAATAT TTTGAAGGACCACCTTTGATTGTAA
**Construction Knock-Out Cassette** ***neoR, natI, Sh Ble***		
23	TEF_R + kanMX (ov)	CGTATAAATCAGCATCCATTCTATATACTGTATACC TTAGTTTCTATGGTTATACCGCTA
24	TEF (ov) + kanMX_L	AGCGGTATAACCATAGAAACTAAGGTATACAGTAT ATAGAATGGATGCTGATTTATACGG
25	kanMX_R + pRS415 (ov)	GCTGGAGCTCCACCGCGGTGGCGGCCGCTCTAGAA CTAGTTAATAAATTATTTTTATTGT
26	kanMX_R + ATFdown (ov)	GGATCGTAAAGTCTATTAAAACTTTTAAAGTAATT GAACTTAATAAATTATTTTTATTGT
27	kanMX (ov) + ATFdown_L	TTCTTGCTTTATAAATAACAACAATAAAAATAATTT ATTAAGTTCAATTACTTTAAAAGT
28	TEF_R + clo (ov)	CCAATGTTTCAGCAACTTGTTCAGGAATAACAGAAA TTTTTCTATATACTGTATACCTTA
29	clo_L + TEF (ov)	AGCGGTATAACCATAGAAACTAAGGTATACAGTAT ATAGAAAAATTTCTGTTATTCCTGA
30	TEF_R + Zeo (ov)	GAGCAGTCAGGACTGGAACAGCAGAGGTGAGTTTA GCCATTCTATATACTGTATACCTTA
31	Zeo_L + TEF (ov)	AGCGGTATAACCATAGAAACTAAGGTATACAGTAT ATAGAATGGCTAAACTCACCTCTGC
32	ATFdown_L + Zeo (ov)	GTTTTATTATCTATTTATGCCCTTATATTCTGTAACTA TCTCAAAATATTGTATTTTCTT
33	Zeo_R + ATFdown (ov)	TAAAAAGAATAAAACTTTGAAAGAAAATACAATAT TTTGAGATAGTTACAGAATATAAGG
34	Zeo_R + pRS415 (ov)	CTGGAGCTCCACCGCGGTGGCGGCCGCTCTAGAACT AGTGGATAGTTACAGAATATAAGG
**Verification of Gene Deletion in** ***H. uvarum*** **(see Figure 2)**		
35	G1 ATF1 F	ATTCCTGCGCAGTCTTAAGCTT
36	G2 R	CAAATCGCTGAAATGGGTGCT
37	G3 F	CAGGTGCTGGTACTGTTGGT
38	G4 ATF1 R NEW	AGAATCTTTTGACCGAGCATGA
39	ATF1_L	TTAATTAAATGCTTACGCTTTCGGATGTTC
40	HuATF1 R	AAAGGCGCGCCTACAATATTTTGACTAAATGTTAT
41	G5 ATFup-TEF_L	CAAAAGGCAACCATTCCCCC
42	G6 ATFdown-Hyg_R	CTGCCATGGCCAATATTCCA

## Data Availability

Not applicable.

## Supplementary Materials

The following are available online at https://www.mdpi.com/1422-0067/22/4/1943/s1, Figure S1: Alignment of the amino acid sequence HuAtf1 with the two Atfs of Saccharomyces cerevisiae. The conserved WRLICLP motif as well as the HXXXDG active site are shown in boxes, Figure S2: Acetate esters, ethyl esters, and acetic acid produced after three days of fermentation of Müller-Thurgau must with Hanseniaspora uvarum DSM2768 for comparison (DSM2768 Ctrl), DSM2768 #34 (WT/Δ), DSM2768 #40 (Δ/Δ), and DSM2768 #46 (Δ/Δ). Pure must without the addition of yeasts served as a negative control (Must Ctrl). The measured acetate esters are: (a) ethyl acetate [mg/L]; (b) isoamyl acetate [μg/L]; (c) 2-methylbutyl acetate [μg/L]; (d) hexyl acetate [μg/L]; (e) 2-phenylethyl acetate [μg/L]; (f) ethyl propionate [μg/L], (g) ethyl butyrate [μg/L] and (h) acetic acid [g/L]. Acetate ester production was measured via HS-SPME-GC-MS analysis. Data are the mean of three independent experiments ± SEM, 1-way ANOVA, Bonferroni’s multiple comparison test, * *p* < 0.05, ** *p* < 0.01, *** *p* < 0.001, **** *p* < 0.0001. Error bars indicate the standard deviation, Figure S3: Amounts of fatty acid and alcohols detected after three days of fermentation of Müller-Thurgau must with Hanseniaspora uvarum, DSM2768 for comparison (DSM2768 Ctrl), DSM2768 #34 (WT/Δ), DSM2768 #40 (Δ/Δ), and DSM2768 #46 (Δ/Δ). Pure must without the addition of yeasts served as a negative control (Must Ctrl). The measured fatty acid and alcohols are: (a) hexanoic acid [mg/L]; (b) isobutanol [mg/L]; (c) isoamyl alcohol [mg/L]; (d) 2-methyl-1-butanol [mg/L]; (e) hexanol [μg/L] and (f) 2-phenyl ethanol [mg/L]. The amounts of fatty acid and alcohols were measured via HS-SPME-GC-MS analysis. Data are the mean of three independent experiments ± SEM, 1-way ANOVA, Bonferroni’s multiple comparison test, * *p* < 0.05, ** *p* < 0.01, **** *p* < 0.0001. Error bars indicate the standard deviation.

## Abbreviations

MDPI	Multidisciplinary Digital Publishing Institute
*ATF1*	Alcohol acetyltransferase I
*TEF1*	Translational elongation factor EF-1α
bp	Base pair
DNA	Deoxyribonucleic acid
SO_2_	Sulphur dioxide
*kanMX*	Geneticin resistance marker
*hygMX*	Hygromycin resistance marker
ORF	Open reading frame
PCR	Polymerase chain reaction
*PGK1*	3-Phosphoglycerate kinase 1
*FBA1*	Fructose-1,6-bisphosphate aldolase 1
*ATF2*	Alcohol acetyltransferase II
*Hph*	Hygromycin b phosphotransferase
*neoR*	Neomycin-geneticin resistance gene
*natI*	Nourseothricin N-acetyltransferase
*Sh Ble*	Bleomycin resistance protein
acetyl-CoA	Acetyl coenzyme A
YPD	Yeast extract peptone dextrose media
ANOVA	Analysis of variance
TE	Tris-ethylenediaminetetraacetic acid
HCl	Hydrogen chloride
EDTA	Ethylenediaminetetraacetic acid
HPLC	High performance liquid chromatography
VCA	Volatile compound analysis
MWD	Multi-wavelength detector
RID	Refractive index detector
HS-SPME-GC-MS	Headspace-solid phase microextraction gas chromatography mass Spectrometry
GC	Gas chromatography
MS	Mass spectrometry
MPS	Multipurpose sampler
CIS	Cooled injection system
NaCl	Sodium chloride
