# Supplementary material for: Development of Genetic Modification Tools for Hanseniaspora uvarum
Source: Int J Mol Sci. 2021 Feb 16;22(4):1943. doi: 10.3390/ijms22041943 (PMC7920042; doi:10.3390/ijms22041943)
Supplement: Supplementary file 1 [file ijms-22-01943-s001.pdf]

Supplementary

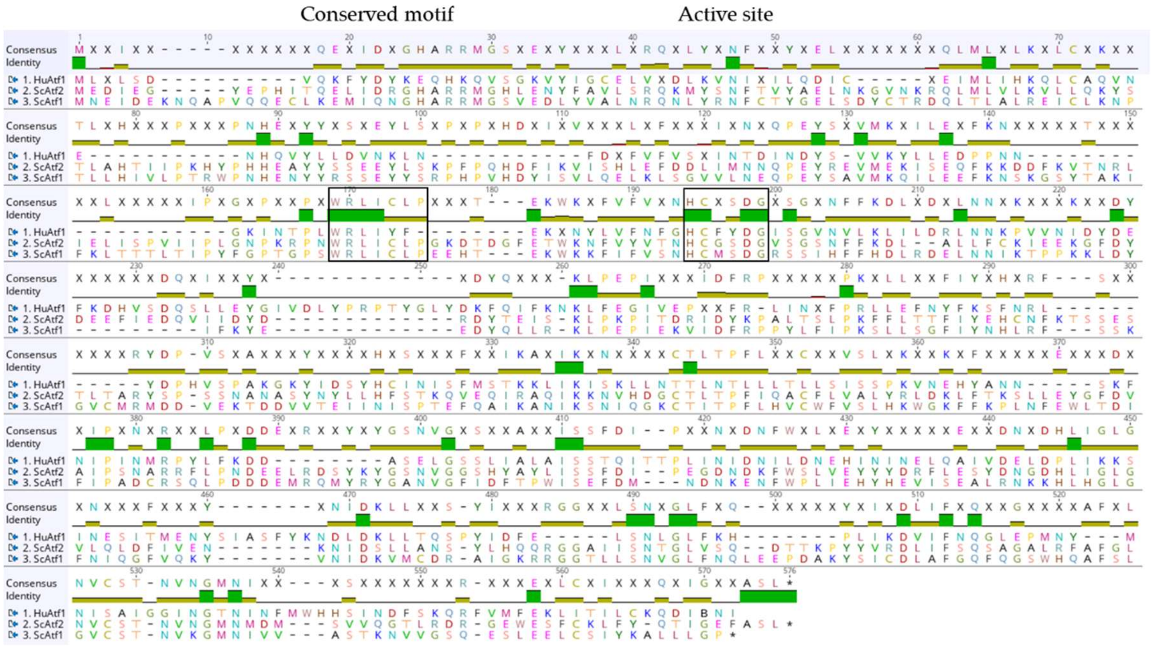

Figure S1. Alignment of the amino acid sequence HuAtf1 with the two Atfs of *Saccharomyces cerevisiae*. The conserved WRLICLP motif as well as the HXXXDG active site are shown in boxes.

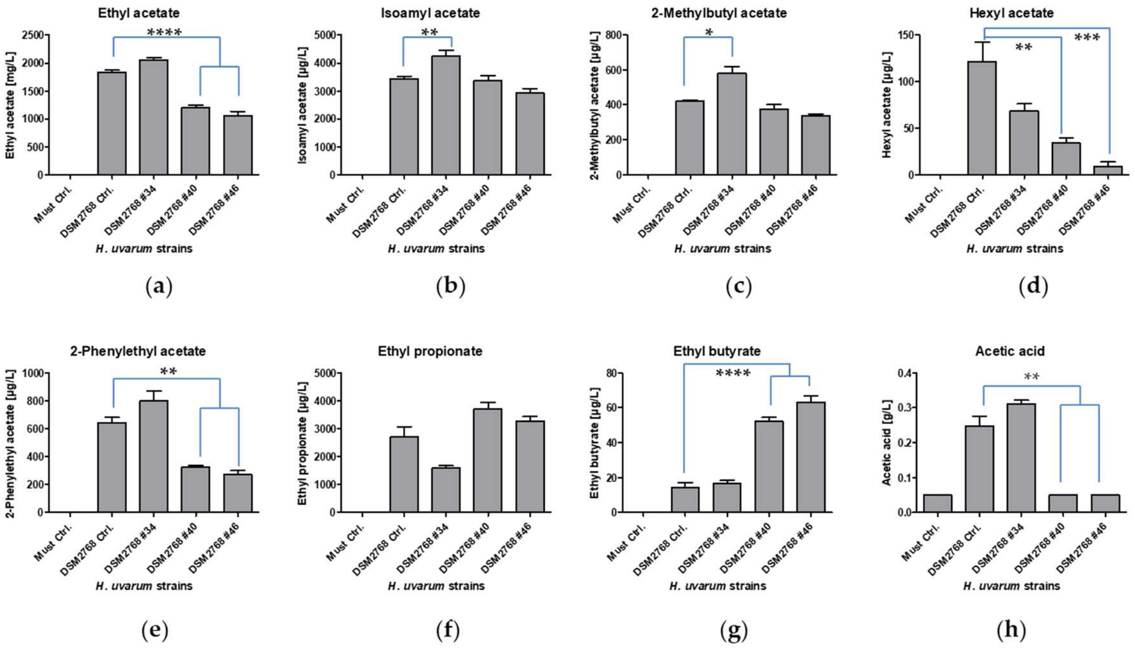

Figure S2. Acetate esters, ethyl esters and acetic acid produced after three days of fermentation of Müller-Thurgau must with *Hanseniaspora uvarum* DSM2768 for comparison (DSM2768 Ctrl.); DSM2768 #34 (WT/Δ); DSM2768 #40 (Δ/Δ) and DSM2768 #46 (Δ/Δ). Pure must without the addition of yeasts served as a negative control (Must Ctrl.). The measured acetate esters are: (**a**) ethyl acetate [mg/L]; (**b**) isoamyl acetate [µg/L]; (**c**) 2-methylbutyl acetate [µg/L]; (**d**) hexyl acetate [µg/L]; (**e**) 2-phenylethyl acetate [µg/L]; (**f**) ethyl propionate [µg/L], (**g**) ethyl butyrate [µg/L] and (**h**) acetic acid [g/L]. Acetate ester production was measured via HS-SPME-GC-MS analysis. Data are the mean of three independent experiments ± SEM, 1-way ANOVA, Bonferroni´s multiple comparison test, *p <0.05, **p <0.01, ***p <0.001, ****p <0.0001. Error bar indicate the standard deviation.

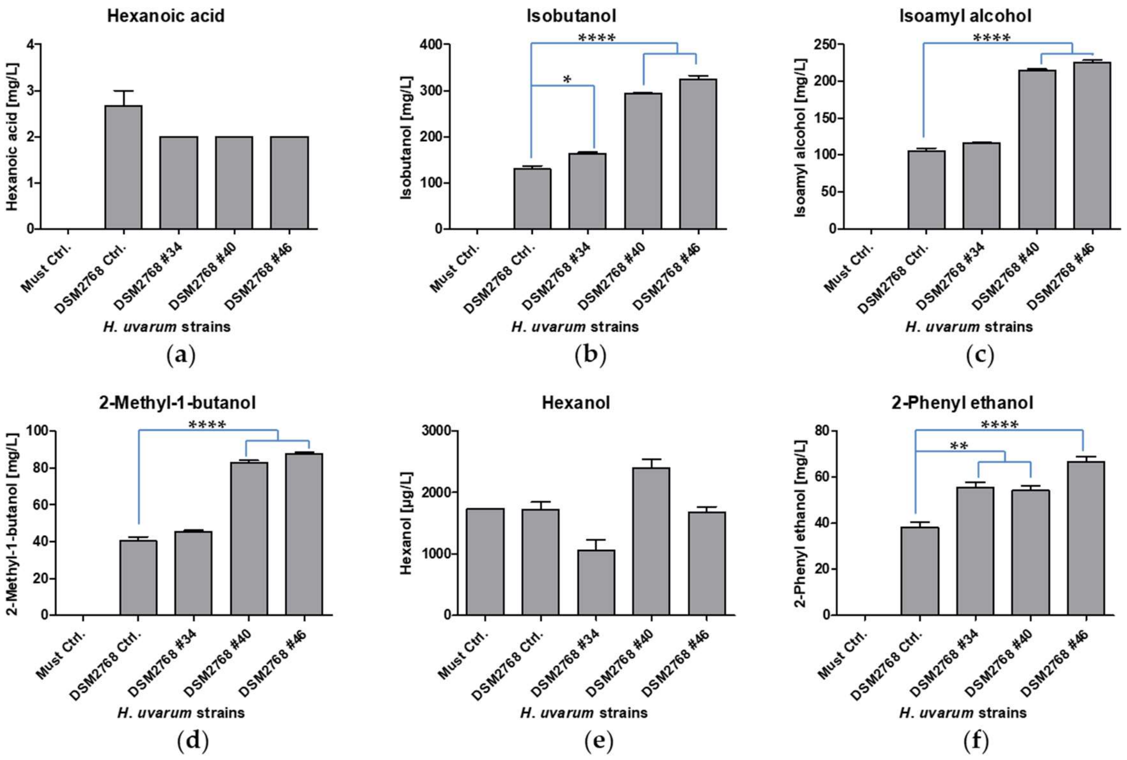

Figure S3. Amounts of fatty acid and alcohols detected after three days of fermentation of Müller-Thurgau must with *Hanseniaspora uvarum* DSM2768 for comparison (DSM2768 Ctrl.); DSM2768 #34 (WT/Δ); DSM2768 #40 (Δ/Δ) and DSM2768 #46 (Δ/Δ). Pure must without the addition of yeasts served as a negative control (Must Ctrl.). The measured fatty acid and alcohols are: (**a**) hexanoic acid [mg/L]; (**b**) isobutanol [mg/L]; (**c**) isoamyl alcohol [mg/L]; (**d**) 2-methyl-1-butanol [mg/L]; (**e**) hexanol [µg/L] and (**f**) 2-phenyl ethanol [mg/L]. The amounts of fatty acid and alcohols were measured via HS-SPME-GC-MS analysis. Data are the mean of three independent experiments ± SEM, 1-way ANOVA, Bonferroni´s multiple comparison test, *p <0.05, **p <0.01, ****p <0.0001. Error bar indicate the standard deviation.
